# 3D printed collimators and dosimetry for spatially fractionated radiation therapy

**DOI:** 10.1002/acm2.70422

**Published:** 2025-12-23

**Authors:** MacKenzie R. Coon‐Haworth, Justin B. Geise, Thirumurugan Elango, Chandler J. Zaugg, Jessica L. Veenstra, Justina J. Riffell, Anna Polkowski, Oluwaseyi M. Oderinde, Judith N. Rivera, Matthew L. Scarpelli

**Affiliations:** ^1^ School of Health Sciences Purdue University West Lafayette Indiana USA; ^2^ Department of Medical Physics Precess Medical Derivatives, Short Hills New Jersey USA; ^3^ Indiana University School of Medicine Indiana University Indianapolis Indiana USA

**Keywords:** Monte Carlo, 3D printing, spatially fractionated radiation therapy, TOPAS

## Abstract

**Background:**

Spatially fractionated radiation therapy (SFRT) has shown incredible potential in sparing normal tissues and activating mechanisms of tumor control distinct from conventional radiotherapy. However, the optimal spatial configuration of SFRT as well as the optimal peak and valley dose values have not been established. This poses a barrier to widespread clinical implementation and efficacy.

**Purpose:**

To facilitate greater SFRT optimization, this work establishes a simple, readily customizable, and cost‐effective approach for fabricating SFRT collimators with different spatial configurations as well as peak and valley dose values.

**Methods:**

The approach involves fabrication of custom SFRT collimators, each consisting of a 3D‐printed plastic shell filled with tungsten. Once fabricated, the collimator dosimetry is characterized using a combination of Gafchromic film and ion chamber measurements. Monte Carlo simulations are used to verify SFRT dosimetry and assess positional uncertainties. The collimators are applied in preclinical mouse experiments demonstrating how they can be used to deliver SFRT.

**Results:**

Five collimators were fabricated for use at kilovoltage energies and one collimator was fabricated for use at megavoltage energies. Across all collimators, the peak widths ranged from 1.2 to 10.1 mm and the valley widths ranged from 1.1 to 10.6 mm. For the kilovoltage collimators, the highest peak‐to‐valley dose ratio was 32.4 at the surface and this dropped to 29.5 at 10 mm depth. For the megavoltage collimator, at 95 cm SSD, the peak‐to‐valley dose ratio was 2.7 at 15 mm depth and this dropped to 2.2 at 100 mm depth. In the mouse experiments, out of multiple SFRT parameters, the mean tumor valley dose had the strongest correlation with change in tumor volume (*p* = 0.02). The Monte Carlo simulations indicated a 5 mm translation of the mouse tumor relative to the beam led to a 44.8% change in the mean tumor dose, underlying the importance of precise positioning for SFRT.

**Conclusions:**

This work establishes a novel approach for custom 3D printing of SFRT collimators and their subsequent characterization. The developed collimators are capable of SFRT delivery at both kilovoltage and megavoltage photon beam energies. This approach facilitates patient specific customization as well as optimization of the peak and valley doses for more effective SFRT.

## 1 INTRODUCTION

Spatially fractionated radiation therapy (SFRT) has been shown to provide tumor control with decreased healthy tissue toxicity.^1^, ^2^, ^3^, ^4^ As a result of its normal tissue sparing, SFRT has become a useful tool for shrinking large bulky tumors. The benefits of SFRT may be derived from its unusual, nonuniform, radiation dose distributions, consisting of high “peak” doses and low “valley” doses.^5^, ^6^, ^7^ ^(chaps1, 17)^ Despite the promise of SFRT, there are major barriers hindering widespread clinical implementation. This includes a gap in knowledge relating to optimal spatial arrangement of the SFRT doses as well as optimal values for the peak and valley doses. Optimization is currently limited as methods for delivering SFRT require equipment not typically available for preclinical research or are too inflexible and expensive for efficient clinical optimization.^8^

Conventional methods of delivering SFRT include the use of multi‐leaf collimators (MLCs) or machined metal blocks.^9^, ^10^ MLC‐based SFRT is highly adaptable, but suffers from extended beam times and is not available on some irradiation systems.^11^ Machined blocks benefit from shorter beam times but are relatively expensive to manufacture and, due to the nature of their production, are not convenient to undergo design iterations.^11^

The goal of the current research was to overcome the implementation challenges associated with SFRT to facilitate optimization and more effective clinical translation. The following describes a readily customizable, yet cost‐effective method for fabricating and characterizing SFRT collimators. The collimators are fabricated for both kilovoltage and megavoltage photon beams, demonstrating the versatility of the approach. Example applications are provided, showing how the collimators can be used to better understand the effects of SFRT. This research provides the initial steps toward optimization of SFRT, paving the way for future improvements in SFRT clinical care.

## 2 MATERIALS AND METHODS

### 2.1 SFRT collimator fabrication

**FIGURE 1:**
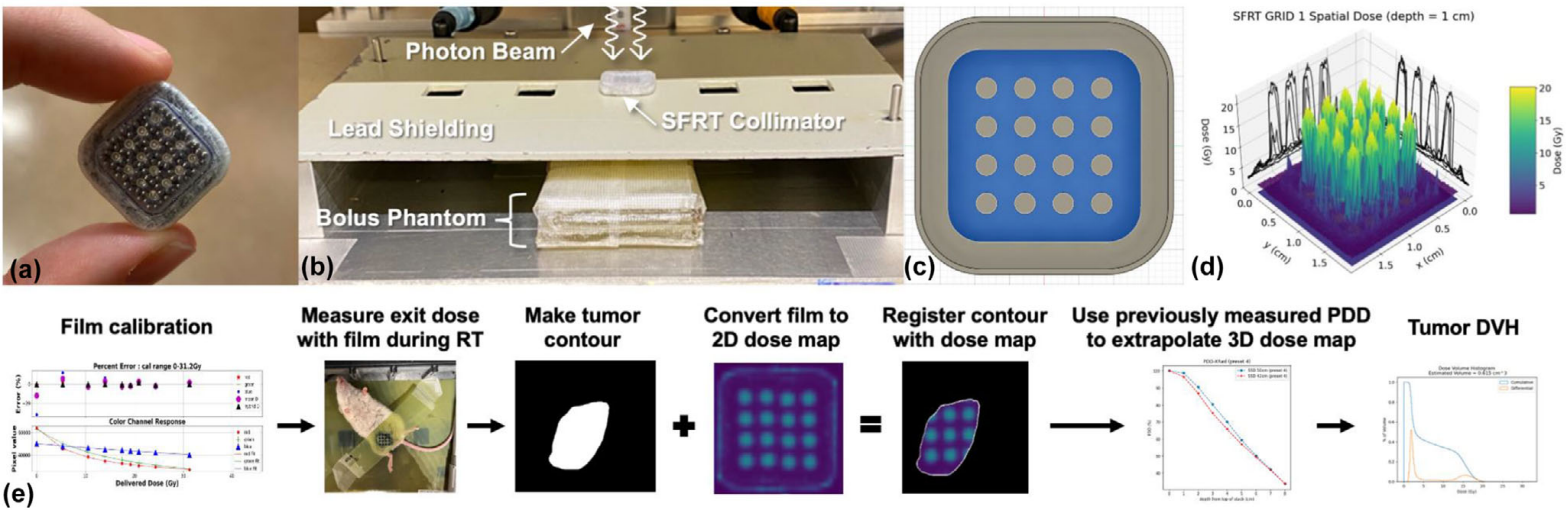
(a) The 3D printed KV GRID 1 collimator filled with tungsten and used in the mouse SFRT experiments. (b) The preclinical SFRT irradiation setup with the bolus phantom underneath the collimator. Additional shielding was added to cover the openings to the left and right. (c) The computer aided drawing of the 3D printed collimator scaffolding. The blue‐shaded area indicates the percent closed area and the grey circles indicate the percent open area. (d) The rendered 3D dose map of KV GRID 1 at 1 cm phantom depth. (e) Film workflow for mouse dosimetry.

The developed fabrication technique utilizes 3D printing, allowing for a novel combination of the benefits of both machining and MLC based techniques. Collimators were designed, each consisting of a plastic shell with plastic pillars acting as “holes” to deliver peak doses. Computer‐aided drawings of these designs can be observed in Figures S1–S9. The shell is filled with commercially available tungsten as the primary compensator material to create valley doses. This includes either 1‐mm diameter tungsten ball bearings (Figure 1a), tungsten grain, or tungsten powder. The selection of which material composition was used as the primary compensator is based on the geometric features of each GRID design, with an appropriate material composition to provide a conformal fill of the plastic shell. Six collimators were designed with this technique and printed with a Formlabs 3BL resin or Creality CR‐10 S5 filament printer. Their geometric parameters and materials are shown in Table 1. The largest advantage of this technique is the ability to reuse the primary attenuator material which decreases the cost of iterating through many designs to optimize design parameters.

**TABLE 1:** SFRT collimator material and geometric design specifications.

SFRT collimator identifier	Scaffold material	Tungsten composition	Primary attenuator thickness (mm)	Pillar diameter (mm)^*^	Pillar spacing (mm)^**^	Pillar pattern	Field size (cm)	Percent open area^***^
KV GRID 1	Formlabs Biomed Clear V1	1 mm Ball Bearings	5.0	1.6	2.8	Square	1.4 x 1.4	16.9%
KV GRID 2A	Overture PLA+	60 Mesh Powder	7.5	3.5	4.25‐4.45	Honeycomb	1.4 x 1.4	40.9%
KV GRID 2B	Overture PLA+	60 Mesh Powder	7.5	2.0	4.25	Honeycomb	1.4 x 1.4	13.4%
KV GRID 3A	Overture PLA+	60 Mesh Powder	7.5	2.1	2.85	Honeycomb	1.4 x 1.4	36.8%
KV GRID 3B	Overture PLA+	60 Mesh Powder	7.5	1.6	2.85	Honeycomb	1.4 x 1.4	21.4%
MV GRID 1	Overture PLA+	Grain	28.5	10	20	Square	16 x 16	15.0%

* Pillar Diameter implies the designed pillar diameter except for the MV GRID 1 design. The MV GRID 1 was designed to achieve a 1 cm peak width at 100 cm SAD, accounting for the beam divergence of the LINAC. Because of this, the pillar diameter varies from top to bottom of the collimator.

** Pillar Spacing implies center‐to‐center pillar spacing for the designs.

*** Visualization of percent open area versus percent closed area is in Figure 1c.

### 2.2 Dosimetry

#### 2.2.1 Kilovoltage collimator characterization

Dosimetric characterization of each collimator consists of measurement of the 2D dose profile, percent depth dose, and output factor. The primary technique used for the dosimetric characterization of the kilovoltage collimator designs is the hybrid triple channel film dosimetry technique^12^ which is implemented using Python scripts. The film used is Gafchromic EBT3 and EBTXD film, depending on the expected dose. To calibrate each batch of film, a NIST traceable cylindrical farmer chamber is used following TG‐61 guidelines.^13^ All films are scanned using an Epson V600 scanner in transparency mode at 300 DPI and saved in a 48‐bit TIFF format. Films were digitized after 24 h + 4Δ*t* using the “one‐scan protocol” as recommended in existing literature.^14^, ^15^ To obtain the dose distributions, a phantom consisting of alternating layers of film and 5 mm Superflab bolus (Radiation Products Design, Inc., Albertville, Minnesota, USA) was irradiated with a variety of x‐ray beam times. This bolus product is similar to solid water and assumed to be tissue equivalent within 3% based upon work by Gargett, et al. in the 300 kV range for low to medium beam qualities^16^ The SFRT collimators are placed directly above the phantom within the 15 × 15 mm^2^ hole in the lead shielding shown in Figure 1b. Irradiation was delivered using an X‐Rad 320 (Precision X‐Ray Inc., North Branford, Connecticut, USA) set to 320 kV, 12.5 mA, and utilizing 2 mm aluminum beam filtration. After the film measurements are converted to two‐dimensional dose maps, the information is used to generate percent depth dose distributions (PDD) and output factors based on the irradiation time.

#### 2.2.2 Megavoltage collimator characterization

Megavoltage compatible collimators were characterized using film dosimetry conducted with a solid water phantom. A Varian EDGE (Varian Medical Systems, Palo Alto, California, USA) at a nominal 6 MV beam energy and dose rate of 600 MU/min was used for the MV GRID 1 characterization. In this case, the collimator is placed on an acrylic photon compensator tray. The film dosimetry measurements were performed at 95 cm SSD for depths of 1.5, 5, and 10 cm.

Several metrics are extracted to characterize the dose distributions created by all collimators at measured depths (Figure 1d). These metrics include peak dose, valley dose, peak width (width at full‐width half‐maximum), valley width, peak‐to‐peak distance, dose rate, and peak‐to‐valley dose ratio (PVDR). The PVDR was calculated using the average of the peaks divided by the average of the valleys.

#### 2.2.3 Mouse dosimetry

To verify doses delivered to mouse subjects, the exit dose or surface dose is measured using film and the dose to the treated volume can be extrapolated from the phantom PDD measurements. To verify the dose to specific structures, such as a tumor, in the absence of image guidance, a contour mask is drawn in GNU Image Manipulation Program (GIMP) that is co‐registered with the dose map to approximate the projection of the structure onto the film based on anatomic markers drawn on the film, caliper measurements, and images. This contour mask is then extruded through space to approximate the structure volume within the treated volume, allowing for extraction of approximated structure specific dosimetry information. From this data, the mean dose, average peak dose, average valley dose, dose–volume histogram (DVH), and equivalent uniform dose (EUD) for the tumor volume can be estimated. Figure 1e illustrates the steps of this workflow.

For mouse tumor dosimetry, EUD was calculated as described in Kuperman et al.^17^ using Equation (1), where fi represents the fraction of the tumor receiving each dose Di. Values of *α* (0.0758) and *β* (0.0918) were calculated based on data from clonogenic assay experiments conducted in previous experiments in our lab.

(1)

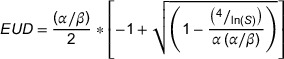

where 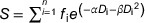 and 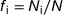.

### 2.3 Computational simulations

#### 2.3.1 Overview

Monte Carlo simulations were used to verify both the dosimetry as well as provide an estimate of dosimetry uncertainties due to tumor positioning. In this study, the Geant4 application toolkit TOPAS MC is used for the Monte Carlo simulation.^18^, ^19^ X‐Rad 320 was designed in TOPAS with the physical and geometrical parameters provided by the manufacturer. It comprises components such as virtual photon beam source, 2 mm aluminum filters, primary and secondary jaws, monitor chamber, mirror at 45⁰ angle along the source to axis distance (SAD), and various water phantoms used for measuring doses. The cabinet of the irradiator was included in the simulation parameters to account for the scattered dose. The virtual photon beam spectrum was simulated using SpekPy V2.0.^20^ The parameters used in the SpekPy included a tungsten target, 30° anode angle, 3 mm beryllium inherent filter, 2 mm aluminum filter, 320 kVp tube voltage, and 12.5 mAs tube current which is the maximum voltage and current setting of the irradiator. ‘Kqp’ physics model and NIST photon dataset were used to generate the spectrum with high accuracy.^20^ Spectrum results were converted into the TOPAS parameter file and included as a virtual photon beam source in the simulation. Source position, focal spot size, and angular spread were matched to the actual machine parameters. Standard EM physics option 4 “g4em‐standard_opt4” and low energy physics list “g4em‐Livermore” are used to achieve accurate dose scoring of orthovoltage x‐ray beams. For every simulation 15 billion histories were used. An electron cut of 0.01 mm is used to increase the efficiency of the simulation while compromising little accuracy.^21^, ^22^

#### 2.3.2 Experimental validation

To characterize and validate the simulation model, experimental HVL and PDD's were compared with the simulated results. The same phantom geometry and dose measurement volume used in the experiment was simulated in the TOPAS. HVL was measured by 0.6 cm^3^ ion chamber in air for a field size of 10 × 10 cm^2^ at 50 cm SSD by introducing 0.635 mm copper plates in the beam. Similarly, PDD was obtained by scoring the dose along the central axis and normalizing the depth dose curve to the maximum dose in a 30 × 30 × 30 cm^3^ water phantom at 50 cm SSD and 10 × 10 cm^2^ field size. Further, the 3D‐printed collimator STL file was imported into the simulation to characterize the grid by comparing the simulated PDD, profiles and peak‐to‐valley dose ratio (PVDR) with the physical measurement results.^23^, ^24^ A 2 mm lead shield was introduced around the grid similar to the experiment for the purpose of shielding the dose outside the grid. A 10 × 10 × 10 cm^3^ phantom was used to score the lateral profile at a certain depth. The PDD was obtained by scoring the dose in the same phantom along the holes in the grid and normalizing the curve to the maximum dose.

#### 2.3.3 Assessment of tumor dosimetry

After validating the simulation model, it was used for analyzing tumor dose distributions. A microCT of a mouse with a tumor was pre‐processed in SlicerRt and imported into TOPAS.^25^ To convert the HU's in the CT to material density, ‘HUtoMaterialSchneider’ parameter file provided by TOPAS was used.^26^ DoseToMedium scorer in TOPAS was used to score the dose.^23^ SlicerRt was used for analyzing the dose distribution results from simulation. Differential DVH (dDVH) from the simulated dose distribution in the tumor was compared to the dDVH from experimental measurements. Uncertainty due to translational and rotational shifts were simulated to understand how the SFRT dosimetry was affected by positional shifts in the tumor.

### 2.4 Mouse mammary tumor model and irradiation

#### 2.4.1 Cell culture

The murine mammary cancer cell line, 4T1, was purchased from American Type Culture Collection (ATCC, Manassas, Virginia, USA) and cultured in RPMI‐1640 (Corning, 10‐041‐CV) base media with 10% fetal bovine serum (FBS, Corning, 35‐010‐CV) and 1% penicillin/streptomycin solution (p/s, Corning, 30‐002‐CI). The cells were maintained in T‐75 culture flasks (Corning, 430641U) in a humidified incubator at 37°C and 5% CO_2_. Once cells reached 70%–80% confluency, they were detached via trypsinization (Corning, 25‐052‐CI) and used for tumor inoculation. Preparation of the cell suspension for inoculation requires the cells to be centrifuged and suspended in phosphate buffered saline (PBS, Corning, 21‐040‐CV) two separate times before counting on a hemocytometer, diluting with PBS to obtain the proper concentration, and placing on ice for injection.

#### 2.4.2 Tumor inoculation and cohort assignments

All animal work is done under a protocol approved by the University Institutional Animal Care and Use Committee (IACUC, ID: 2203002258A008). Twelve‐week‐old BALB/cJ mice (*n* = 15) (Jackson Laboratories, 000651) were inoculated orthotopically in the lower right mammary fat pad with 4T1 cells. The tumor cell concentration was 2 × 10^6^ cells/mL, with each injection consisting of 1 × 10^5^ cells. All mice were humanely anesthetized using 1%–3% concentration vaporized isoflurane in oxygen gas for the inoculation procedure as is approved in our IACUC protocol.

Tumors were allowed to grow untreated until the majority reached at least 200 mm^3^, after which they underwent irradiation. Mouse weight and tumor measurements were taken daily. Equation (2) was used to estimate the tumor volume from caliper measurements. All mice were euthanized on the same day and tumors were excised for analysis.

(2) $$TumorVolume\left(mm^{3}\right)=\frac{\pi }{6}\left(length\right)\left(width\right)\left(height\right)$$

#### 2.4.3 Tumor irradiation

Radiotherapy of mouse tumors was carried out using an X‐Rad 320 (Precision X‐Ray Inc., North Branford, Connecticut, USA) preclinical cabinet irradiator with peak energy set to 320 kV, current of 12.5 mA, and 2 mm aluminum beam filtration. The treatment table was moved to 30 cm from the beam source, yielding open beam dose rates up to 6.4 Gy/min. The SFRT was delivered as a single fraction of radiation with a planned tumor volume mean dose of 20 Gy. This corresponded to 626 s of beam time. The KV GRID 1 collimator was used and it was placed within the hole of the lead shielding above the mouse (Figure 1b). During the irradiation procedure, mice were anesthetized with cocktail of 27% ketamine and 2.4% xylazine in saline, injected interperitoneally. After treatment, mice were monitored until they recovered from anesthesia.

#### 2.4.4 Imaging

The mice were imaged utilizing the Quantum GX microCT system (PerkinElmer, Inc.). Whole‐body CT images were acquired prior to radiotherapy for use in the TOPAS Monte Carlo simulation.

#### 2.4.5 Mouse brain irradiation and γH2AX

To assess the spatial pattern of DNA damage after SFRT, a single C57BL/6J mouse was orthotopically injected with 5 × 10^5^ GL‐261 cells. Seven days later, the mouse was anesthetized and irradiated with a single 20 Gy mean dose to the whole brain using the same setup as described above with the KV GRID 1 collimator. Within 10 min after radiation was administered, the mouse was euthanized, and the brain was fixed in a 4% paraformaldehyde solution. 24 h following fixation, the brain was placed in 70% ethanol and shipped in 70% ethanol. Automated immunohistochemistry analysis staining for γH2AX was performed by HistoWiz Inc. (Brooklyn, NY).

## 3 RESULTS

### 3.1 Dosimetric characterization of SFRT collimators

Averaged values from film dosimetry characterization of the SFRT collimators were obtained and are tabulated in Table 2. The KV GRID 1 collimator was measured at an additional source‐to‐table distance of 30 cm as the mice were treated at this distance rather than the default of 50 cm.

**TABLE 2:** Dosimetric characterization of 3D printed SFRT collimators. Displayed values are mean ± standard deviation.

SFRT collimator identifier	SSD (cm)	Beam time (sec)	Phantom depth (mm)	Avg peak dose (Gy)	Avg valley dose (Gy)	Avg PVDR	Avg peak dose rate (Gy/min)	Avg peak width (mm)	Avg valley width (mm)	Avg peak‐to‐peak distance(mm)
KV GRID 1	Source to Table = 30	300	0	30.8 ± 0.3	2.0 ± 0.1	15.8 ± 0.9	6.2 ± 0.1	1.8 ± 0.0	1.2 ± 0.2	3.0 ± 0.2
		300	10	22.4 ± 0.3	2.2 ± 0.1	9.9 ± 0.6	4.5 ± 0.1	1.8 ± 0.1	1.2 ± 0.0	3.0 ± 0.2
	Source to Table = 50	950	0	25.1 ± 0.3	3.1 ± 0.1	8.2 ± 0.2	1.6 ± 0.0	1.7 ± 0.0	1.2 ± 0.0	2.9 ± 0.1
		950	10	19.7 ± 0.4	3.3 ± 0.2	6.0 ± 0.3	1.3 ± 0.0	1.7 ± 0.1	1.2 ± 0.1	2.9 ± 0.2
KV GRID 2A	Source to Table = 50	1000	0	30.8 ± 0.7	2.1 ± 0.2	14.4 ± 1.6	1.6 ± 0.6	2.9 ± 0.1	1.3 ± 0.1	3.8 ± 0.8
		1000	10	23.9 ± 0.6	2.6 ± 0.3	9.2 ± 1.1	1.3 ± 0.5	3.0 ± 0.1	1.3 ± 0.1	4.3 ± 0.8
KV GRID 2B	Source to Table = 50	1000	0	26.2 ± 1.2	0.8 ± 0.1	32.4 ± 2.7	1.4 ± 0.5	1.6 ± 0.0	2.6 ± 0.1	4.1 ± 0.3
		1000	10	20.4 ± 0.9	0.7 ± 0.1	29.5 ± 2.8	1.1 ± 0.4	1.6 ± 0.1	2.6 ± 0.1	4.3 ± 0.5
KV GRID 3A	Source to Table = 50	1000	0	23.4 ± 1.3	1.4 ± 0.1	16.6 ± 1.6	1.4 ± 0.1	1.7 ± 0.1	1.1 ± 0.1	2.8 ± 0.4
		1000	10	17.8 ± 1.0	1.8 ± 0.2	10.0 ± 1.2	1.1 ± 0.1	1.7 ± 0.1	1.1 ± 0.1	2.8 ± 0.3
KV GRID 3B	Source to Table = 50	1000	0	22.0 ± 1.6	0.9 ± 0.1	24.8 ± 2.8	1.3 ± 0.1	1.2 ± 0.2	1.5 ± 0.1	2.7 ± 0.2
		1000	10	17.0 ± 1.3	1.0 ± 0.1	16.5 ± 1.9	1.0 ± 0.1	1.2 ± 0.2	1.5 ± 0.1	2.8 ± 0.4
MV GRID 1	95	3500 MU, 350 sec	15	33.3 ± 0.5	12.4 ± 0.5	2.7 ± 0.9	5.7 ± 0.1	9.3 ± 0.1	9.8 ± 0.1	19.2 ± 0.6
			50	27.4 ± 0.3	10.8 ± 0.5	2.5 ± 0.6	4.7 ± 0.0	9.6 ± 0.1	10.1 ± 0.1	19.8 ± 0.5
			100	20.4 ± 0.5	9.4 ± 2.6	2.2 ± 0.2	3.5 ± 0.1	10.1 ± 0.2	10.6 ± 0.2	20.8 ± 0.9

The SFRT geometric parameters measured from the irradiated films were similar to the designed collimator dimensions in Table 1. The percentage difference data comparing the designed versus measured values is listed in Table S1. The mean percent difference between the measured peak‐to‐peak width and designed pillar spacing was −0.5% (range −10.8% to 8.2%). When comparing the peak width with the designed pillar diameter, the mean percent difference was −10.7% (range −28.5% to 12.9%). The valley width corresponds to the designed edge‐to‐edge spacing of the pillars. The mean percentage difference between valley width and designed edge‐to‐edge spacing was 15.1% (range −2.5%to 52.7%). Most of the differences can be attributed to the amount of attenuating material between the pillars, the thickness of attenuating material, and beam divergence.

For collimator peak dose and peak dose rate, the values were as expected with higher dose delivered at the surface than at depth. Valley doses for each collimator had mixed trends. Collimators KV GRID 1, KV GRID 2A, KV GRID 3A, and KV GRID 3B had higher valley doses at 10 mm depth than at the surface, which may be due to increased scatter from the peak to the valley regions. KV GRID 2B and MV GRID 1 had decreased valley doses at greater depths. This is likely due to the relatively low percent open area of these two collimators relative to the other collimators, limiting scatter from the peak to the valley regions.

For all collimators, the PVDR were greater at the surface compared to depth. The largest PVDR was for KV GRID 2B with a value of approximately 30. This is attributed to the relatively low percent open area of that collimator and the lower energy of the x‐rays, limiting the scatter interactions in the peak regions. The PVDR was the lowest for MV GRID 1 with an approximate value of 2.7 at 15 mm depth. This is due to the higher beam energy used for MV GRID 1, the increased field size, and a larger open area of the collimator.

**FIGURE 2:**
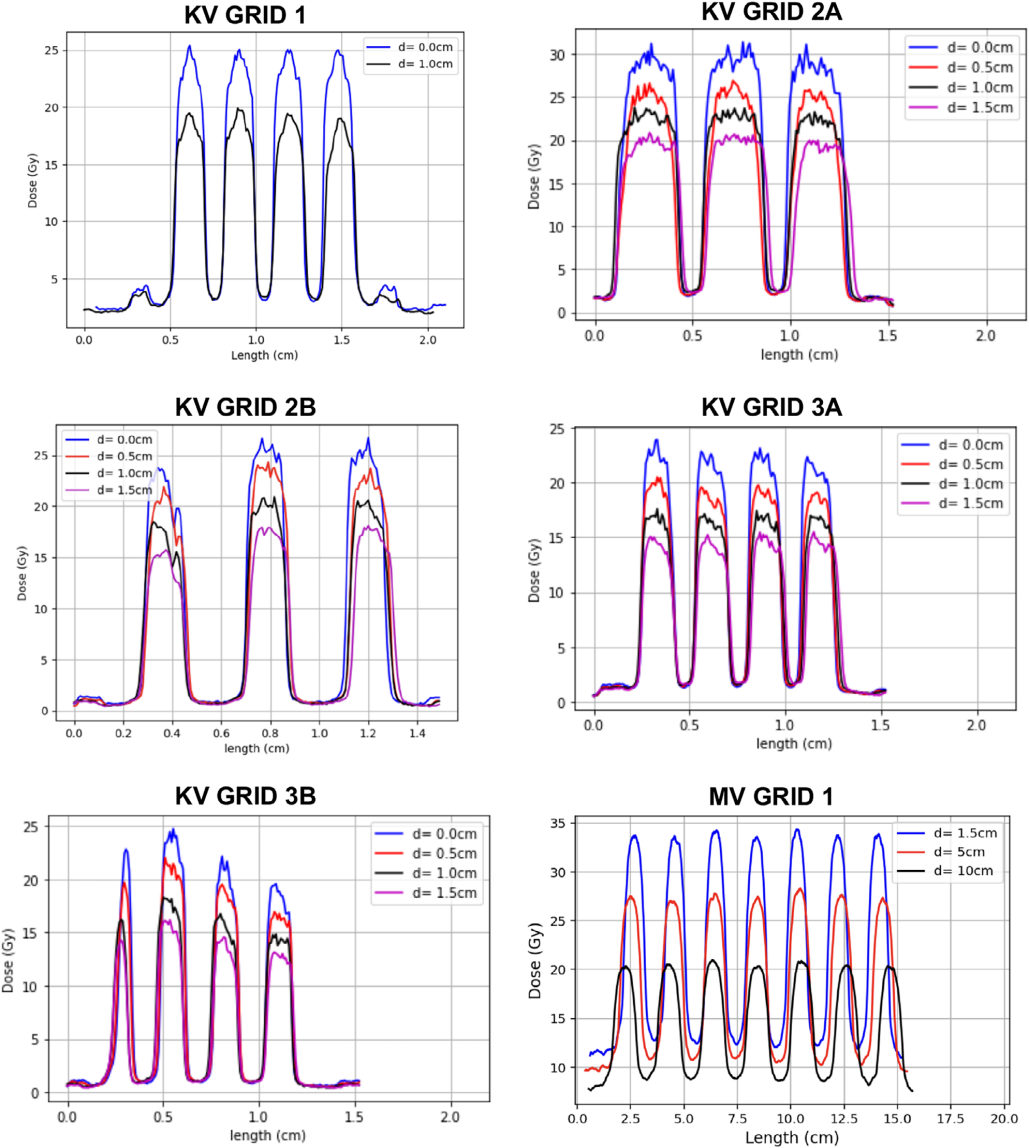
Graphs show dose profiles obtained from film measurements along a horizontal line through a representative row of each collimator design. Dose values along this line were averaged vertically over a width of 0.0762 cm. KV GRID 2A, 2B, 3A and 3B correspond to an irradiation time of 1000 s; KV GRID 1 corresponds to 950 s; and MV GRID 1 corresponds to 350 s.

A visual representation of the collimator depth dose curves can be observed in Figure 2. There is greater uniformity in the KV GRID 1 peak doses than in the other KV collimators. This is likely due to it being printed using a superior 3D printer and material than the other KV grids. KV GRID 3B has the greatest variation in the peak doses, which is visually apparent in Figure 2 and in the standard deviations of Table 2. This is likely due to it having the smallest pillar size, which was approaching the lower limit of the capabilities of that particular 3D printer and scaffold material.

### 3.2 Monte Carlo simulations

#### 3.2.1 Dosimetric characterization of the X‐Rad 320 irradiator with open field

**FIGURE 3:**
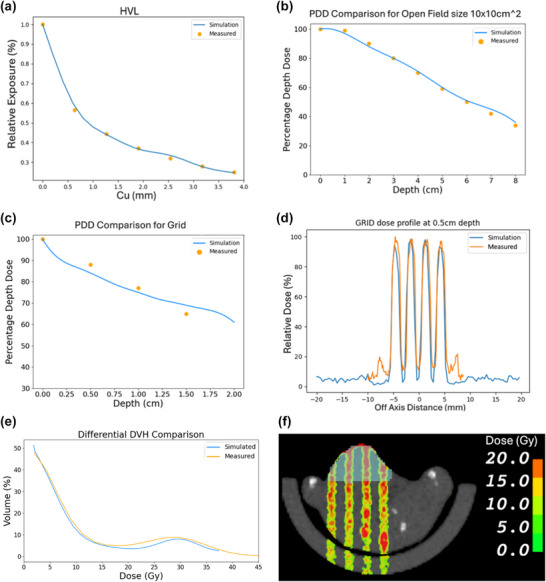
(a) Comparison plot between simulated and measured HVL. (b) Comparison plot between simulated and measured PDD in a 10 x 10 cm^2^ Field size. (c) Comparison plot between simulated and measured PDD in KV GRID 1. (d) Comparison of simulated and measured collimator profile at 0.5 cm depth in a phantom. (e) Comparison of differential DVH between simulated and measured doses. (f) Simulated dose distribution for KV GRID 1 in a mouse mammary tumor.

The relative exposure for both experiment and simulation for various thickness of copper plates is plotted in Figure 3a. The average difference between the two curves is 0.57%. The half value layer, with no SFRT collimator, was found to be 0.935 mm of copper from experiment and 1.008 mm of copper from simulation. Figure 3b represents the comparison between experiment and simulation of the PDD for an open 10 × 10 cm^2^ field size at 50 cm SSD. The measured PDD was found to have a good agreement with the simulated PDD, with a maximum difference of 3.0%.

#### 3.2.2 Dosimetric characterization of the KV GRID 1 SFRT collimator

Figure 3c shows the comparison of measured and simulated PDD's at 50 cm SSD along the holes in the KV GRID 1 collimator. Measured and simulated PDD with the collimator had good agreement with a maximum difference of 4.1%. Figure 3d shows the dose profile of the simulated and measured KV GRID 1 collimator at 0.5 cm depth in a 30 × 30 × 30 cm^3^ phantom. The peak dose has a maximum difference of 3.6% between measurement and simulation. The peak FWHM is 1.62 mm from simulation and 1.70 mm from measurement. Mean peak‐to‐peak distance is 2.90 mm from measurement and 2.95 mm from simulation. Valley doses have a maximum difference of −6.0% between measurement and simulation. The underestimation of the measured valley dose is likely due to several factors adding to the inability of the Monte Carlo model to accurately calculate the doses in the valley region.^27^ First, when the SFRT collimator was imported into TOPAS as an STL file, the plastic resin encapsulating the tungsten was also assigned to its material density equivalent to tungsten. Additionally, while tungsten ball bearings were used in the experimental setup, the simulated collimator was filled with solid tungsten material, which does not accurately reflect the experimental conditions. Furthermore, discrepancies between the geometry of the 3D‐printed and the CAD‐designed collimator arising from limitations in 3D printing technology may have also contributed to variations in the simulation. A solution to this problem includes assigning resin to its respective material density instead of tungsten.

#### 3.2.3 Dosimetric characterization of the mouse mammary tumor

Figure 3f shows the simulated dose distribution for KV GRID 1 in a mouse mammary tumor. For visualization, the dose calculated from TOPAS is overlayed on the CT scan of the mouse mammary tumor. The green segmented region in the CT represents the tumor volume. The average dose, average peak dose, average valley dose to the segmented tumor was found to be 9.6, 29.9, and 3.5 Gy from simulations. The percentage difference between simulated and measured dosimetry for this particular tumor was as follows: average tumor dose −1.4%, average peak dose + 3.5%, and average valley dose −7.4%. Furthermore, at 0.5 cm depth, the PVDR from simulation was found to be 8.6, while the measured PVDR was 7.7.

To further investigate the dose distribution within the tumor, the differential DVH was plotted for experimental and simulated results (Figure 3e). The average difference and maximum difference between both curves were found to be 6.4% and 16.1%, respectively. The maximum difference was found in the low dose region of the curve. The percentage of tumor covered by the peak dose in measurement and simulation was 23.5% and 41.8%, respectively. This relatively large difference is due to positional errors in the experimental setup. In the simulated case, the tumor was placed at the center of the KV GRID 1, whereas in the measured case, the tumor was inadvertently placed slightly off center. This led to a decrease in peak coverage and emphasizes the importance of correct alignment for SFRT.

#### 3.2.4 Dose uncertainty due to positional shifts

Given the relatively large deviation between simulated and measured percent tumor in the peak region, mentioned above, we performed a simulation assessment of how positional uncertainties of the mouse tumor influence the SFRT dosimetry. The results are listed in Table 3 for KV GRID 1. The average tumor dose deviates minimally when a 2 mm translational shift is applied to the tumor; however, when a 5 mm translational shift is applied to the tumor, the average tumor dose decreases by almost 50%. This is because lateral shifts of 5 mm caused the field to miss some of the tumor volume, hence the average dose to the tumor is reduced. Less variation was evident during rotational shifts. However, with rotational shifts, the dose distribution varied significantly in deeper depths distal to the tumor. The tumor in this case was seated superiorly; hence, the effect of the rotational shift was not significant.

**TABLE 3:** The various mouse orientations and their effect on the SFRT dose distribution for KV GRID 1.

Orientation of mice	Peak dose volume (%)	Mean tumor dose (Gy)	Mean tumor dose difference (%)	Mean peak dose (Gy)	Mean valley dose (Gy)	Gamma comparison
No shifts	41.8 (0.159cc)	9.6	NA	29.9	3.5	NA
5‐degree rotation	34.2 (0.130cc)	9.6	0.0	29.5	5.4	90.8
10‐degree‐rotation	32.15 (0.122cc)	9.5	−1.0	29.6	4.7	85.9
15‐degree‐rotation	32.3 (0.123cc)	8.5	−11.5	28.6	3.4	83.2
2 mm lateral translation	39.2 (0.149cc)	9.4	−2.1	28.9	4.2	0.1
5 mm lateral translation	6.03 (0.023cc)	5.3	−44.8	31.0	2.8	50.2

The gamma comparison between the reference dose and 2 mm lateral translation is close to zero because the peak dose of reference dose matches with the valley dose region of the translated dose distribution and vice versa. However, this may not be the case for other SFRT collimators where peaks and valleys are spaced differently.

### 3.3 Application in mouse mammary tumor model

#### 3.3.1 Correlation of dosimetric parameters with tumor control

**FIGURE 4:**
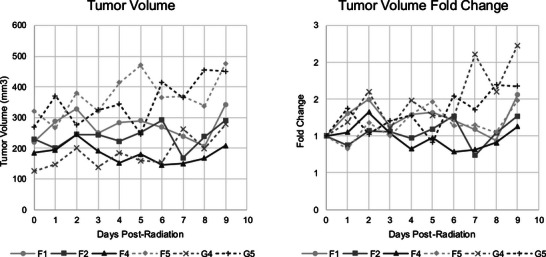
Tumor volume estimates (left) and fold change (right) from the daily caliper measurements after radiation treatment.

The KV GRID 1 SFRT collimator was applied in a mouse mammary tumor model to demonstrate how the developed dosimetric methods can be used to evaluate SFRT. Tumor volumes and normalized fold change of the volumes for all SFRT treated mice are plotted in Figure 4. Tumor volumes were measured daily until endpoint criteria, which occurred nine days after irradiation, due to tumor cavitation (IACUC‐designated endpoint criteria).

Post hoc analysis of the film dosimetry for the SFRT‐treated mouse tumors yielded several estimates of dose received by each mouse as shown in Table 4. Additionally, the bottom half of Table 4 shows Pearson correlation coefficients that were calculated to determine the correlation between these variables and tumor control, shown with their corresponding *p*‐value. Tumor volume at the time of radiotherapy and the mean tumor dose did not have a significant correlation with fold change in tumor volume for SFRT‐treated mice. Volume average peak dose within the tumor for the SFRT mice showed a strong positive correlation with fold change in tumor volume, approaching statistical significance (*p*‐value = 0.06). Volume average tumor valley dose showed a significant strong negative correlation with fold change in tumor volume (*p*‐value = 0.02). Both the total volume average dose and the EUD had a weak negative correlation with no significance.

**TABLE 4:** Comparison of radiation dose parameters for SFRT treated mice and correlation of radiation dose parameters to relative change in tumor volume.

Mouse ID	Tumor volume at RT (mm3)	Volume average peak dose (Gy)	Volume average valley dose (Gy)	Total volume average dose (Gy)	EUD (α = 0.0758, *β* = 0.0918) (Gy)	Percent of contour in peak dose (%)
F1	219.8	45.4	7.4	18.1	6.6	24.2
F2	229.1	45.8	7.1	17.4	6.4	21.8
F4	184.7	45.0	7.1	18.1	6.3	24.3
F5	321.4	47.7	7.3	16.3	5.5	19.3
G4	124.6	45.3	6.4	17.1	5.9	22.4
G5	268.6	48.0	6.6	16.4	5.6	22.0
Correlation with fold change in tumor volume at endpoint						
Pearson Correlation Coefficient (*p*‐value)	−0.41 (0.41)	0.79 (0.06)	−0.85 (0.02)	−0.23 (0.67)	−0.39 (0.44)	−0.12 (0.82)

#### 3.3.2 Assessment of SFRT induced DNA damage in vivo

**FIGURE 5:**
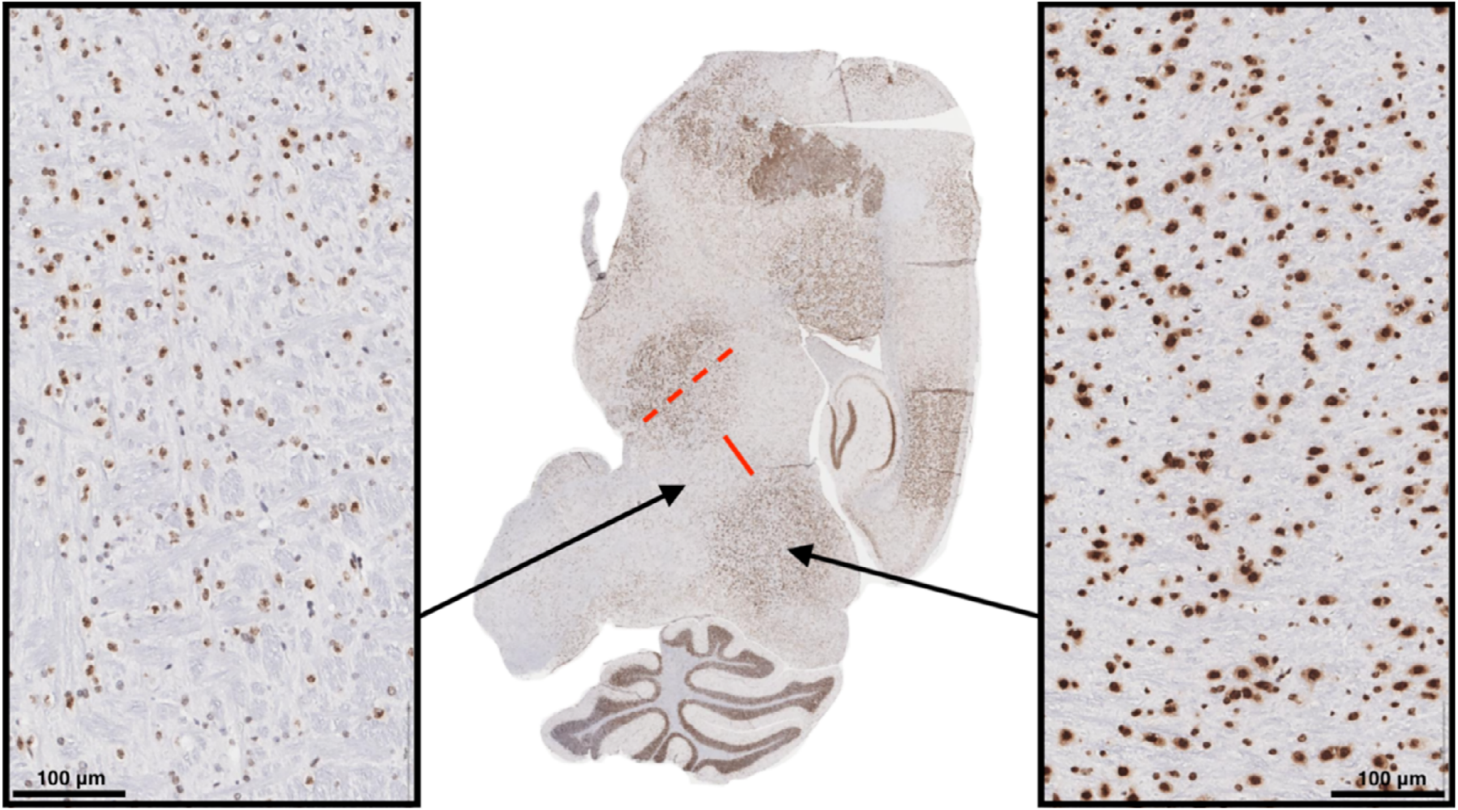
Sagittal section of the mouse brain stained with γH2AX to visualize DNA damage. The middle panel shows the raw immunohistochemistry image, with the dark regions indicating sites of double‐strand breaks. The left panel shows a magnified view of the valley region, while the right panel shows a magnified view of the peak dose region. The dotted red line indicates the diameter of the peak dose region, and the solid red line shows the valley width. An implanted mouse brain tumor is evident in the top of the whole brain image with intense γH2AX staining relative to the surrounding normal tissue.

To verify the in vivo spatial patterns of SFRT, we assessed the DNA damage pattern for KV GRID 1. For this application, a mouse was irradiated with a mean prescription dose of 20 Gy to the brain in a single fraction. The brain was resected, and sagittal brain sections were stained using γH2AX to visualize DNA double‐strand breaks. As shown in Figure 5, the spatial patterns of SFRT are observable with intense positive staining in the peak regions and low intensity staining in the valley regions. Using the mean intensity measured by QuPath,^28^ the peak‐to‐valley γH2AX ratio (PVR) was calculated to be 2.4. The diameter of the high intensity γH2AX regions is approximately 1.7 mm, which is similar to the average peak width of 1.8 mm measured with film dosimetry for KV GRID 1 (Table 2 ). The average valley width of the low intensity γH2AX regions was 0.8 mm, which is smaller than the 1.2 mm valley width measured with film dosimetry for KV GRID 1 (Table 2).

## 4 DISCUSSION

This study establishes a novel approach for fabrication and characterization of SFRT collimators. This approach has some advantages over prior techniques. It eradicates the need for metal machining of grids for delivering SFRT. Additionally, the fabrication method allows for custom collimator designs to be created in a cost and time‐effective manner. The collimators are mechanically stable and have withstood repeated use in our experiments, but their longevity is an avenue of future investigation. This approach facilitates systematic investigation of many collimators and SFRT parameters. Given SFRT incorporates many dosimetric parameters not used in conventional radiotherapy, there is a need to identify which of these parameters have the most relevance to outcome.^7^ ^(chap17),^ ^29^, ^30^, ^31^ The approach here enables fabrication of SFRT collimators with a range of parameter values and the subsequent determination of which parameter values are optimal for tumor control and normal tissue sparing. This optimization would facilitate more effective SFRT treatment and greater clinical translation.

Similar approaches have been used to create electron cutouts for personalized patient treatment.^32^, ^33^ In the referenced methods,^32^, ^33^ a cutout template was printed with plastic filament such as PLA, which was then filled with tungsten ball bearings. The main difference from the current study is this prior work was used for electron beams rather than photon beams. Others^34^ have used molds created from plastic sheets filled with tungsten ball bearings as IMRT compensators on linear accelerators and Co‐60 based machines. This method did not utilize 3D printing or SFRT but did use their tungsten‐filled devices for radiotherapy. Another unique method utilized 3D printing to fabricate SFRT collimators with tungsten doped PLA filament.^35^ The printed SFRT collimator was able to fit in a 9.3 × 9.3 × 7.1 cm^3^ block containing a hole pattern based on the design by Nobah et al.^36^ While this method proved to be precise and effective, it requires a printer with a nozzle capable of printing a metallic doped material to minimize filament irregularities when deposited on the print bed, which could affect print resolution and increase costs. Alternatively, in the method presented here, by 3D printing the scaffolding and filling with tungsten, nearly any 3D printer can be utilized and the tungsten could be removed and reused for future design iterations, making it relatively affordable and accessible.

Monte Carlo simulations can save resources by simulating various SFRT collimators and their effects on tumor dosimetry. Hence, in this study we aimed to characterize and validate an SFRT simulation model by comparing it with experimental measurements. Once validated, the simulation was used to investigate the SFRT dose distribution in a mouse mammary tumor.^22^, ^23^, ^24^ Each individual simulation required around 8.5 h of CPU time on Intel i7‐13700 processor. Though current simulation time is a limiting factor, it still provides some useful insights. The simulation revealed positional uncertainties in a mouse tumor relative to the SFRT collimator can lead to significant changes in the tumor dose distribution. Specifically, the mean tumor dose decreased by > 40% with a 5 mm lateral shift in the mouse tumor relative to the SFRT collimator. This necessitates having some method for verifying delivered doses on a subject‐by‐subject basis to ensure SFRT prescription doses are achieved. The film dosimetry method outlined here offers one solution for doing this in preclinical experiments.

Immunohistochemical analysis using γH2AX staining qualitatively confirmed that the spatially modulated dose distribution is produced in vivo in mouse brain tissue. Peak dose regions showed intense staining for DNA double‐strand breaks, while the valley dose regions had a much lower intensity, providing an in vivo representation of what was observed in the film dosimetry. The PVR derived from γH2AX staining was 2.4, which is notably lower than the PVDR of approximately 10 calculated from the collimator's dose measurements. This discrepancy could be explained by the fact that yH2AX foci overlap at higher radiation doses, leading to signal saturation and a plateau in peak yH2AX intensity.^37^

The spatial dimensions of the γH2AX peak regions matched film dosimetry, but the measured γH2AX valley width was narrower than film dosimetry. One possible explanation for this discrepancy is that geometric misalignment between the radiation beam and the tissue slicing plane may have caused distortion. Another potential explanation is bystander effects, wherein radiation from the peak regions causes DNA damage in surrounding valley regions. Regardless, this finding suggests that while the overall spatial pattern of SFRT is preserved in tissue, factors such as orientation and slicing plane require careful consideration. This would be essential for the accurate interpretation of the in vivo spatial effects in future SFRT studies.

Analysis of subject‐specific film dosimetry for mouse mammary tumors provides valuable insight into the dose distributions of SFRT, including their subsequent impact on tumor response. The strong negative correlation between the SFRT tumor valley dose and fold change in tumor volume underscores the potential significance of low‐dose regions in SFRT, possibly contributing to enhanced therapeutic efficacy through mechanisms such as microvascular disruption or immune modulation.^29^, ^38^ It is important to note that while tumor control is a crucial measurable outcome, some of the benefits of SFRT, such as immune modulation, normal tissue sparing, and microvascular changes, are not always directly observable without invasive techniques and that is an area of future investigation.

## 5 CONCLUSION

Overall, these findings present a relatively simple and cost‐effective approach for fabricating SFRT collimators. This approach can be used in future studies incorporating biological markers and mechanistic evaluations, which will be critical to refining our understanding of SFRT and optimizing its therapeutic potential. Future investigations should also address subject‐specific tumor positioning and field design to minimize unintended dose discrepancies and improve consistency in SFRT delivery. These future studies should aim to establish the optimal SFRT parameters for tumor control and normal tissue sparing. This would further increase utilization of SFRT and improve care of cancer patients with large tumors.

## AUTHOR CONTRIBUTIONS

All authors contributed to the final manuscript via discussion, review, and/or writing. MacKenzie R. Coon‐Haworth conceived and fabricated the initial preclinical kilovoltage 3D printed collimator design, conducted mouse mammary model experiments, and obtained CT images of mice. Justin B. Geise conceived and fabricated the megavoltage 3D printed collimator design and generated the python script for film calibration and analysis, as well as conducted megavoltage collimator characterization. Thirumurugan Elango planned and conducted all simulations and uncertainty analysis. Thirumurugan Elango, Justin B. Geise, and MacKenzie R. Coon‐Haworth contributed to statistical analysis. MacKenzie R. Coon‐Haworth and Justin B. Geise and Justina J. Riffell generated data from scanned films using the python script, conducted film measurements of the kilovoltage collimators, carried out film calibrations and collimator characterizations. Justin B. Geise., Chandler J. Zaugg, Oluwaseyi M. Oderinde, and Judith N. Rivera performed the megavoltage collimator measurements and data collection. Chandler J. Zaugg and Jessica L. Veenstra conducted the mouse brain SFRT experiment. Justina J. Riffell and Anna Polkowski created and printed a vast majority of the kilovoltage collimators. Matthew L. Scarpelli directed the study, discussed results, and edited the manuscript.

## CONFLICT OF INTEREST STATEMENT

The authors disclose that there are no conflicts of interest.

## Supporting information



Supporting Information

## Data Availability

The data from this manuscript will be made available upon reasonable request to the corresponding author.
