# Supplementary material for: 3D printed collimators and dosimetry for spatially fractionated radiation therapy
Source: J Appl Clin Med Phys. 2025 Dec 23;27(1):e70422. doi: 10.1002/acm2.70422 (PMC12724578; doi:10.1002/acm2.70422)

Supplemental Table S1: Comparison between the measured parameters from film dosimetry and the designed constraints using percent difference. Due to the divergent nature of the MV GRID design, the "Designed Pillar Diameter" is the designed peak width at a given depth. This same concept applies for the MV GRID "Designed Center-to-Center Pillar Spacing" and "Designed Edge-to-Edge Pillar Spacing." Percent difference is calculated using Supplementary Equation S1.

SFRT Collimator Identifier	SSD (cm)	Beam Time (sec)	Phantom Depth (mm)	Avg Peak Width (mm)	Designed Pillar Diameter (mm)	Percent Difference	Avg Peak-to-Peak Dist (mm)	Designed Center-to-Center Pillar Spacing (mm)	Percent Difference	Avg Valley Width (mm)	Designed Edge-to-Edge Pillar Spacing (mm)	Percent Difference
KV GRID 1	Source to Table = 30	300	0	1.77	1.60	10.09%	2.96	2.80	5.56%	1.17	1.20	-2.53%
		300	10	1.82	1.60	12.87%	3.04	2.80	8.22%	1.24	1.20	3.28%
	Source to Table = 50	950	0	1.70	1.60	6.06%	2.85	2.80	1.77%	1.17	1.20	-2.53%
		950	10	1.70	1.60	6.06%	2.94	2.80	4.88%	1.21	1.20	0.83%
KV GRID 2A	Source to Table = 50	1000	0	2.91	3.50	-18.31%	3.81	4.25	-10.81%	1.27	0.75	51.63%
		1000	10	3.03	3.50	-14.30%	4.26	4.25	0.24%	1.29	0.75	52.65%
KV GRID 2B	Source to Table = 50	1000	0	1.56	2.00	-24.78%	4.14	4.25	-2.67%	2.56	2.25	12.73%
		1000	10	1.60	2.00	-22.35%	4.29	4.25	0.98%	2.63	2.25	15.65%
KV GRID 3A	Source to Table = 50	1000	0	1.69	2.10	-21.58%	2.75	2.85	-3.46%	1.06	0.75	33.98%
		1000	10	1.74	2.10	-18.75%	2.79	2.85	-2.06%	1.09	0.75	36.87%
KV GRID 3B	Source to Table = 50	1000	0	1.20	1.60	-28.49%	2.72	2.85	-4.78%	1.49	1.25	17.38%
		1000	10	1.24	1.60	-25.43%	2.76	2.85	-3.06%	1.52	1.25	19.32%
MV GRID 1	100	3500 MU, 350 sec	15	9.26	9.65	-4.12%	19.22	19.30	-0.42%	9.78	9.65	1.34%
			50	9.60	10.00	-4.08%	19.80	20.00	-1.01%	10.14	10.00	1.39%
			100	10.11	10.50	-3.78%	20.75	20.10	3.18%	10.63	10.50	1.23%

Supplemental Equation S1:

$$Percent\ Difference = \frac{Measured - Designed}{\left(\frac{Measured + Designed}{2}\right)} \times 100\%$$

Computer Aided Drawings of Scaffold Design

Supplemental Figure S1a: Drawing of scaffold design for KV GRID 1 used for 3D printing.

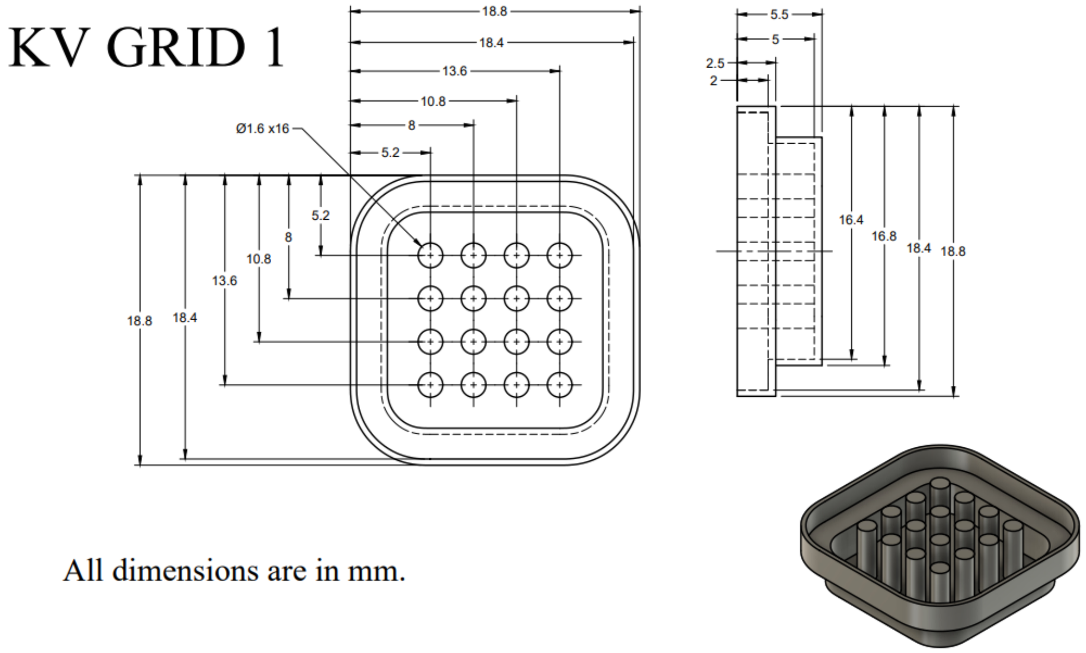

Supplemental Figure S1b: Drawing of scaffold design for KV GRID 2A used for 3D printing.

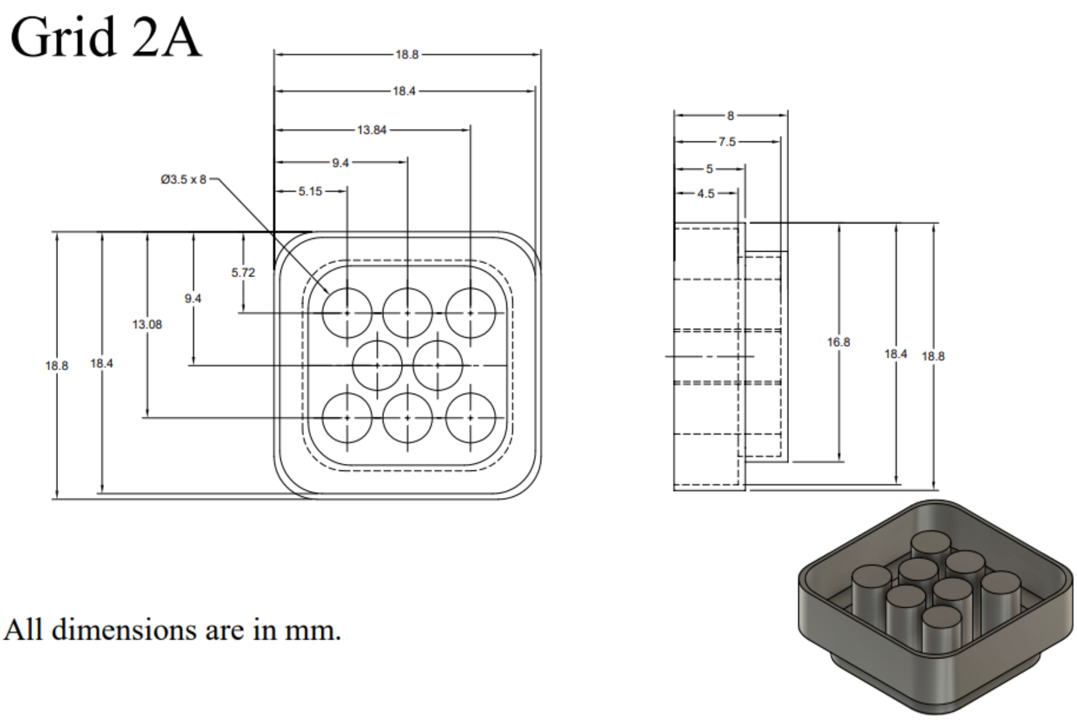

Supplemental Figure S1c: Drawing of scaffold design for KV GRID 2B used for 3D printing.

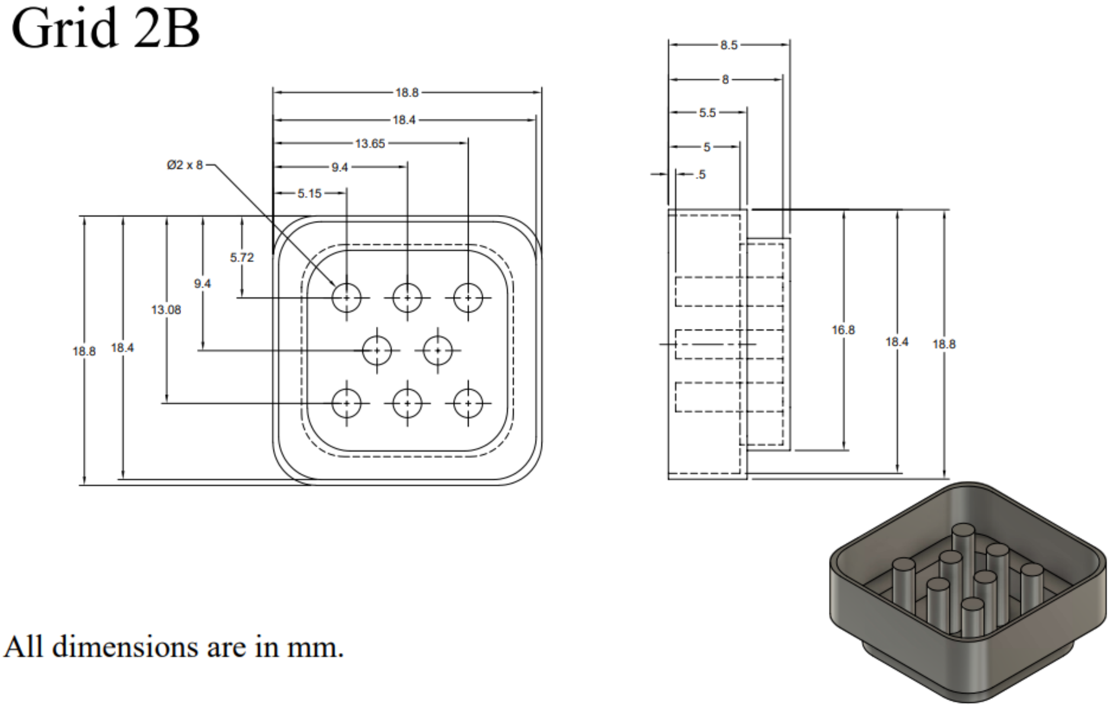

Supplemental Figure S1d: Drawing of scaffold design for KV GRID 3A used for 3D printing.

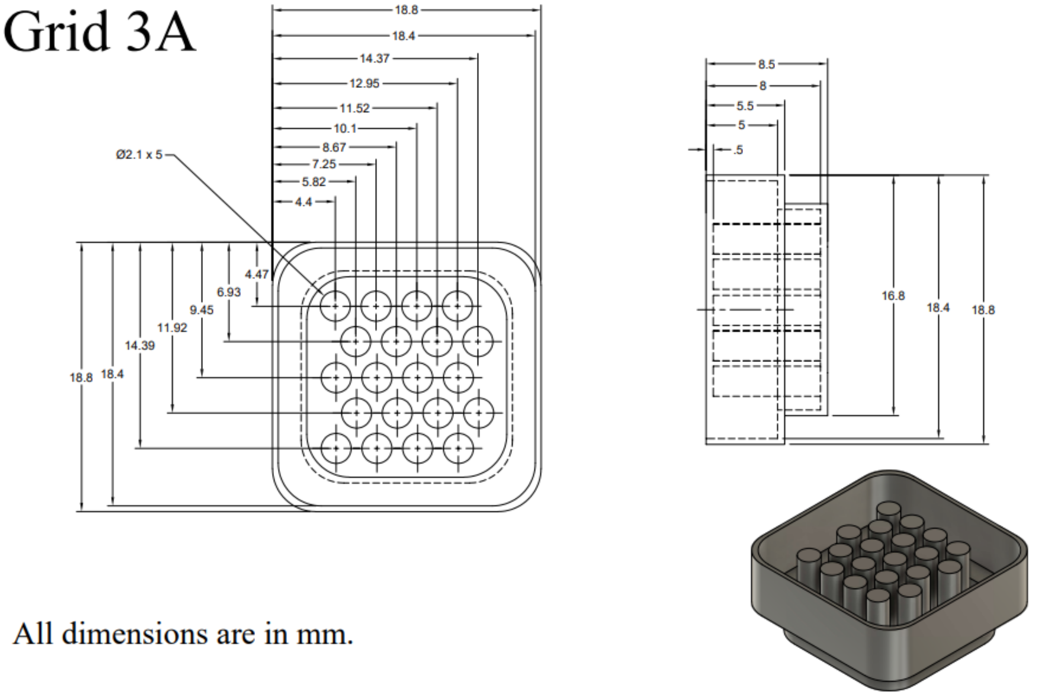

Supplemental Figure S1e: Drawing of scaffold design for KV GRID 3B used for 3D printing.

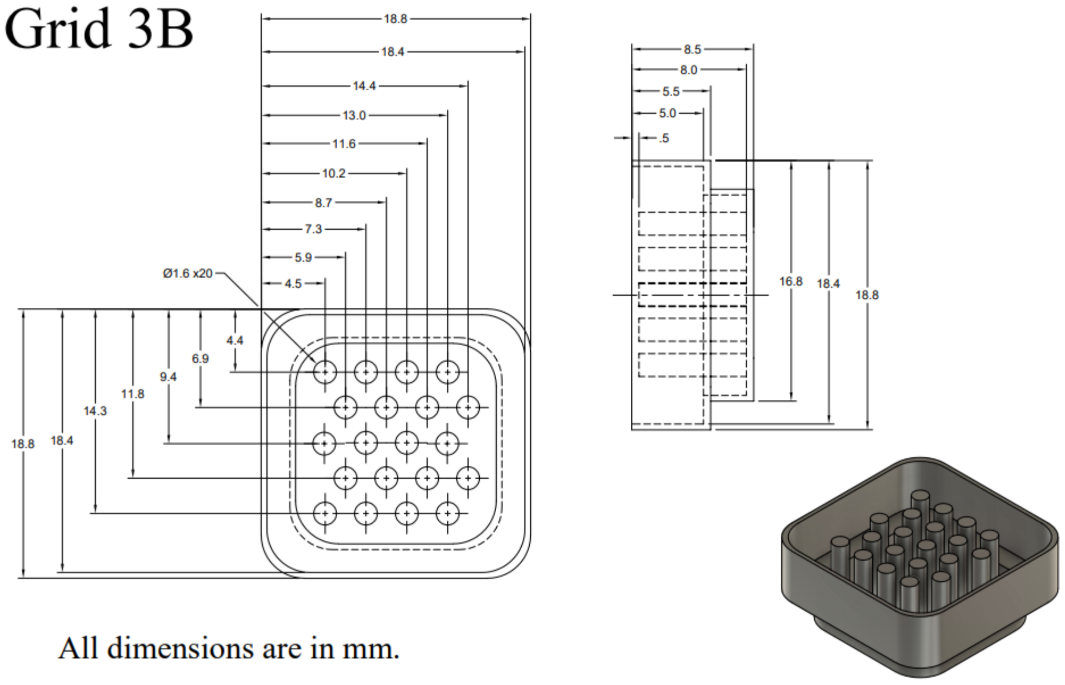

Supplemental Figure S1f: Drawing of assembly design for MV GRID 1 used for 3D printing.

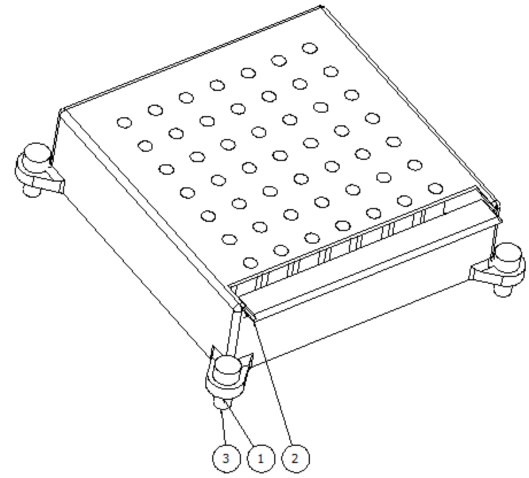

PARTS LIST			
ITEM	QTY	PART NUMBER	DESCRIPTION
1	1	1cm _1cm _diverge_Varian_ Edge	3-D printed collimator shell
2	1	Removeable_lid	Lid for collimator shell
3	4	attachment pin	Pins for attachment to photon compensator tray

Supplemental Figure S1g: Drawing of scaffold design for MV GRID 1 used for 3D printing.

MV GRID 1

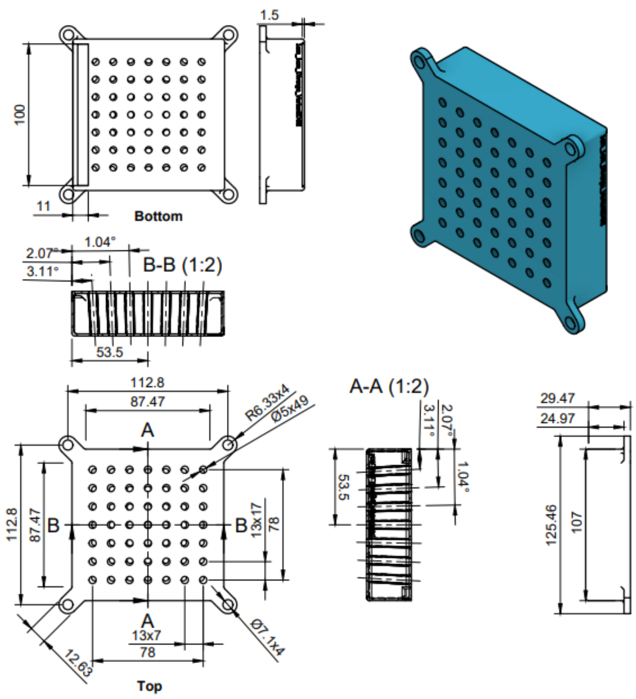

All dimensions are in mm.

Supplemental Figure S1h: Drawing of Attachment Pin for 3D printing used for attaching MV GRID 1 to a photon compensator tray.

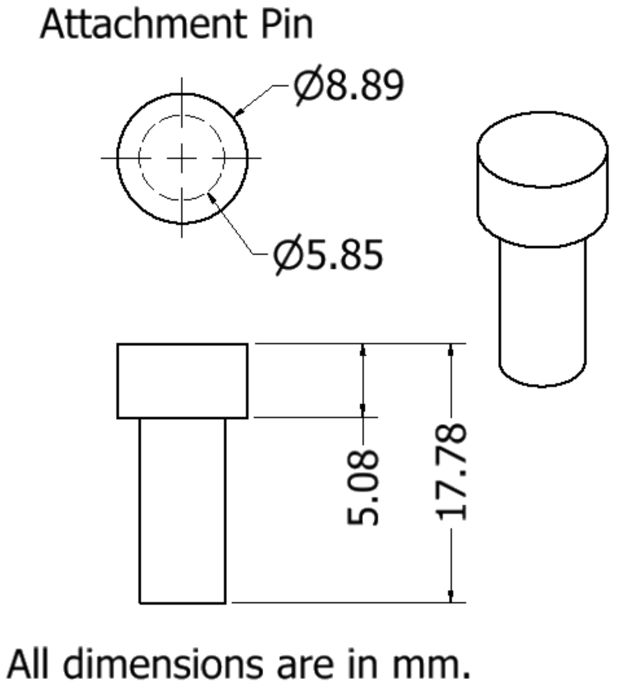

Supplemental Figure S1i: Drawing of Removeable lid for enclosing MV GRID 1 used for 3D printing.

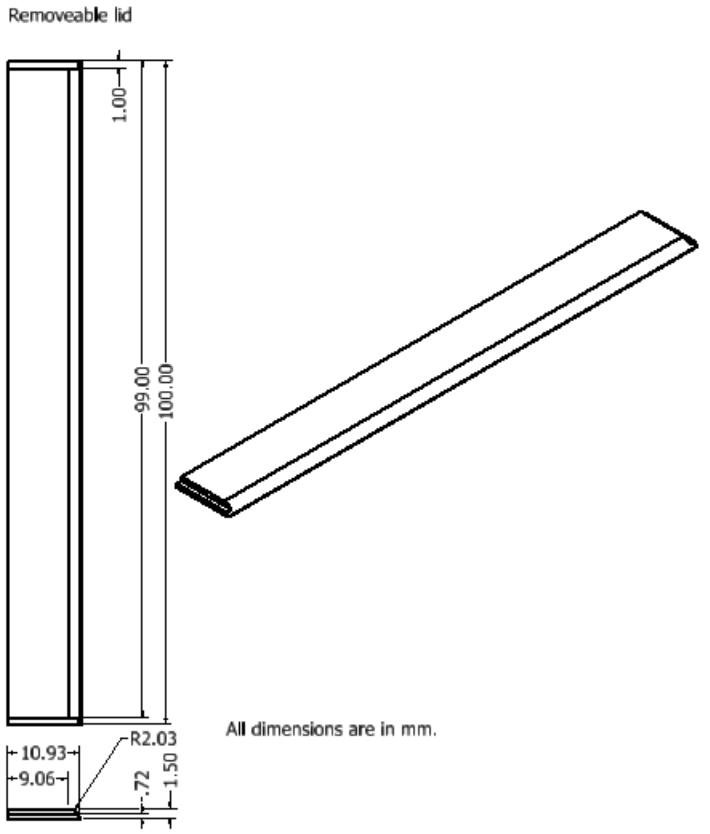

Supplement: Supplementary file 1 — Supporting Information [file ACM2-27-e70422-s001.pdf]
